# Enhancing Working Memory Based on Mismatch Negativity Neurofeedback in Subjective Cognitive Decline Patients: A Preliminary Study

**DOI:** 10.3389/fnagi.2020.00263

**Published:** 2020-09-29

**Authors:** Guangying Pei, Ruoshui Yang, Zhongyan Shi, Guoxin Guo, Shujie Wang, Miaomiao Liu, Yuxiang Qiu, Jinglong Wu, Ritsu Go, Ying Han, Tianyi Yan

**Affiliations:** ^1^School of Life Science, Beijing Institute of Technology, Beijing, China; ^2^School of Mechatronical Engineering, Beijing Institute of Technology, Beijing, China; ^3^Graduate School of Natural Science and Technology, Okayama University, Okayama, Japan; ^4^Faculty of Engineering, Okayama University, Okayama, Japan; ^5^Department of Neurology, Xuanwu Hospital, Capital Medical University, Beijing, China; ^6^Center of Alzheimer’s Disease, Beijing Institute for Brain Disorders, Beijing, China; ^7^National Clinical Research Center for Geriatric Disorders, Xuanwu Hospital, Capital Medical University, Beijing, China

**Keywords:** mismatch negativity, neurofeedback, working memory, subjective cognitive decline, Alzheimer’s disease, early intervention

## Abstract

Mismatch negativity (MMN) is suitable for studies of preattentive auditory discriminability and the auditory memory trace. Subjective cognitive decline (SCD) is an ideal target for early therapeutic intervention because SCD occurs at preclinical stages many years before the onset of Alzheimer’s disease (AD). According to a novel lifespan-based model of dementia risk, hearing loss is considered the greatest potentially modifiable risk factor of dementia among nine health and lifestyle factors, and hearing impairment is associated with cognitive decline. Therefore, we propose a neurofeedback training based on MMN, which is an objective index of auditory discriminability, to regulate sensory ability and memory as a non-pharmacological intervention (NPI) in SCD patients. Seventeen subjects meeting the standardized clinical evaluations for SCD received neurofeedback training. The auditory frequency discrimination test, the visual digital N-back (1-, 2-, and 3-back), auditory digital N-back (1-, 2-, and 3-back), and auditory tone N-back (1-, 2-, and 3-back) tasks were used pre- and post-training in all SCD patients. The intervention schedule comprised five 60-min training sessions over 2 weeks. The results indicate that the subjects who received neurofeedback training had successfully improved the amplitude of MMN at the parietal electrode (Pz). A slight decrease in the threshold of auditory frequency discrimination was observed after neurofeedback training. Notably, after neurofeedback training, the working memory (WM) performance was significantly enhanced in the auditory tone 3-back test. Moreover, improvements in the accuracy of all WM tests relative to the baseline were observed, although the changes were not significant. To the best of our knowledge, our preliminary study is the first to investigate the effects of MMN neurofeedback training on WM in SCD patients, and our results suggest that MMN neurofeedback may represent an effective treatment for intervention in SCD patients and the elderly with aging memory decline.

## Introduction

Alzheimer’s disease (AD) is slow and progresses with a presymptomatic course over several years to decades (Sperling et al., 2011). Controlling modifiable risk factors for AD at the preclinical stage remains the most realistic preventive strategy (Crous-Bou et al., 2017). Subjective cognitive decline (SCD) is a condition that is manifested by healthy older people who show unimpaired performance on cognitive tests and self-report a perceived cognitive decline in memory or other cognitive domains, such as executive function or attention (Rabin et al., 2015). SCD may represent the first symptomatic manifestation of AD before mild cognitive impairment (MCI; Koppara et al., 2015; Bubbico et al., 2019). Currently, there is no consensus on the best intervention or treatment for SCD with regard to psychological, cognitive, lifestyle, and pharmacological interventions described in a recent systematic review and a meta-analysis (Williams and Tanabe, 2016; Bhome et al., 2018). Given the heterogeneity of etiology of SCD and specifically considering individuals who have minimal manifestation of clinical symptoms, it is difficult to confirm a focal target of pharmacological intervention that does not cause an adverse reaction. Therefore, non-pharmacological intervention (NPI) may be a feasible method of treatment for patients with SCD (Smart et al., 2017; Bhome et al., 2018).

Elderly SCD subjects have a subtle decline in memory performance associated with accelerated memory decline, and SCD may predict future objective memory decline, even for incident dementia (Koppara et al., 2015). There is no authoritative method for treating early memory loss; however, a growing number of publications suggests that neurofeedback, which as a form of electroencephalogram (EEG) biofeedback used to self-regulate individual own brain activity, can directly alter the underlying neural mechanism of cognition and behavior (Enriquez-Geppert et al., 2017; Bhome et al., 2018). Neurofeedback has been successfully applied in the treatment of various diseases, such as attention-deficit hyperactivity disorder (ADHD; Van Doren et al., 2019) or epilepsy (Van Doren et al., 2019), enhancing memory for the healthy old adults with aging cognitive decline (Reis et al., 2016), and even in dementia or AD patients (Luijmes et al., 2016; Berman and Nichols, 2019; Kaufmann et al., 2019). For example, AD patients have an excess of slow frequency waves, such as delta and theta, and a reduction in the alpha waves compared with that in healthy aging individuals; thus, regulating the abnormal EEG frequency activity can have a positive effect on clinical performance, especially considering the effect of neurofeedback on cognitive ability (Luijmes et al., 2016). Here, we propose using neurofeedback as a NPI to improve the cognitive ability of SCD patients.

Hearing loss was considered to be the greatest potentially modifiable risk factor for dementia among the nine health and lifestyle factors according to a novel dementia risk model based on lifespan reported by the Lancet Commission (Uchida et al., 2019). If middle-aged hearing loss is eliminated, the risk of dementia may be reduced by 9% (Lin F. R. et al., 2013; Livingston et al., 2017). A 25-year study self-reported that hearing loss is linked to accelerated cognitive decline in older people (Amieva et al., 2015). A meta-analysis suggested that hearing impairment is related to cognitive impairments (Taljaard et al., 2016). Auditory mismatch negativity (MMN) is a negative wave of event-related potential (ERP) typically obtained by the standard stimulus and is subtracted from the deviant stimulus in the oddball tasks (Näätänen and Alho, 1997), with MMN as an objective index of auditory discriminability (Garrido et al., 2009). Electrophysiological studies have shown that when any detectable change in a regular pattern of auditory stimulation occurs, a preattentive change-detection system in the auditory modality emits a signal called MMN (Molholm et al., 2005). Several studies have shown that the MMN peak latency is systematically prolonged and its amplitude is attenuated with aging (Molholm et al., 2005; Näätänen et al., 2011). In various neurological disorders, including schizophrenia, autism spectrum disorders, and dementia, MMN appears to provide an objective tool for investigation of auditory processing and memory trace attenuation (Chen et al., 2017). Recent studies have shown that MMN amplitude in AD patients for a long interstimulus interval (ISI) of 3 s is smaller than that for the shorter ISIs of 1 s, while the amplitude is stable in the control group of healthy subjects (Lindín et al., 2013; Laptinskaya et al., 2018). Moreover, the MMN amplitude was significantly lower in amnestic MCI adults compared with that in healthy people. MMN can be a relatively sensitive psychophysiological biomarker in identifying amnestic MCI (Lindín et al., 2013). Furthermore, auditory working memory (WM) is the major factor of neural processing of sound, and cognitive factors shape the brain networks for auditory skills (Kraus et al., 2012). Auditory training helps to compensate for the degradation of the auditory signals; the training demonstrated that cognitive function can be improved during the index executive process, competing speech, and dual-task performance. Importantly, auditory-cognitive training showed general improvements in speech, auditory WM, and processing speed, as well as enhanced self-reporting of communication difficulties (Sweetow and Sabes, 2006; Ferguson and Henshaw, 2015). At present, MMN neurofeedback has been successfully used in adults mainly for the adjustment of sensory abilities, such as improving auditory discriminability for two particularly similar tones and enhancing language ability and music level (Chang et al., 2014, 2017). Although the improvement of sensory ability can improve work memory by cognitive training, there are insufficient clinical data on optimization of cognitive ability by MMN neurofeedback training; to the best of our knowledge, no studies have investigated the effect of MMN neurofeedback on memory brain function.

In this study, we recruited patients with SCD mainly for the short-term tight MMN neurofeedback training. The present pilot study aims to investigate the neurofeedback training based on MMN amplitude to determine whether the training has a positive effect on the regulation of the MMN characteristic in SCD patients. It is hypothesized that the MMN amplitude can be improved by short-term close training based on neurofeedback. Second, we hypothesized that WM performance may be enhanced by MMN neurofeedback training in patients with SCD. MMN neurofeedback may be an effective intervention method to enhance WM in SCD patients and the elderly with aging memory decline.

## Materials and Methods

### Participants

A total of 17 right-handed patients with memory concerns were recruited from the memory clinic of the Department of Neurology, Xuanwu Hospital, Beijing, China. The study was approved by the Medical Research Ethics Committee and Institutional Review Board of Xuanwu Hospital. All subjects underwent a series of standardized clinical assessments, including a medical history interview, a neurological examination, a blood examination, and a series of neuropsychological tests. All participants provided a written informed consent before performing any experimental procedures. We performed neuropsychological tests to evaluate cognitive function, social and daily functions, mental behavioral symptoms, sleep, clinical characteristics, and various cognitive domain scales. The scale results of the participants are shown in Table 1. Experienced neurologists performed the diagnoses. The diagnosis of SCD was based on published SCD research criteria proposed by the Subjective Cognitive Decline Initiative (SCD-I; Jessen et al., 2014). SCD subjects were able to complete all neurofeedback training within 2 weeks.

**Table 1:** Demographics and characteristics of the sample.

Number	17
Gender (Female/Male)	12/5
Age (year)	64.35 ± 7.86
Education (year)	12.82 ± 4.10
Hand (Left/Right)	0/17
**Cognitive Function (mean ± SD)**	
MMSE (Mini-Mental State Examination)	27.35 ± 1.54
MES (Memory and Executive Screening)	85.24 ± 23.47
MOCA-B (Montreal Cognitive Assessment-B)	25.41 ± 2.67
**Excutive Ability (mean ± SD)**	
STT-A (Shape Trails Test; second)	58.25 ± 17.84
STT-B (Shape Trails Test; second)	146.53 ± 54.58
**Memory (mean ± SD)**	
AVLT-h (Auditory Verbal Learning Test-Hearing; delay)	6.12 ± 2.83
AVLT-h (Auditory Verbal Learning Test-Hearing; recognition)	22.00 ± 1.41
**Language Skill (mean ± SD)**	
BNT (Boston Naming Test)	25.06 ± 3.94
**Social and Everyday Functions (mean ± SD)**	
ECog (Everyday Cognition)	1.50 ± 0.43
**Mental Behavioral Symptoms (mean ± SD)**	
HAMD (Hamilton Depression Scale)	3.71 ± 3.29
HAMA (Hamilton Anxiety Scale)	5.71 ± 5.32
GDS (Geriatric Depression Scale)	2.53 ± 2.21
**Sleep Activity (mean ± SD)**	
PSQI (Pittsburgh Sleep Quality Index)	6.47 ± 4.47
RBDS (Rapid Eye Movement sleep Behavior	1.18 ± 1.42
Disorder Screening Questionnaire)
ESS (Epworth Sleepiness Scale)	6.94 ± 5.04

### Experimental Design

**Figure 1:**
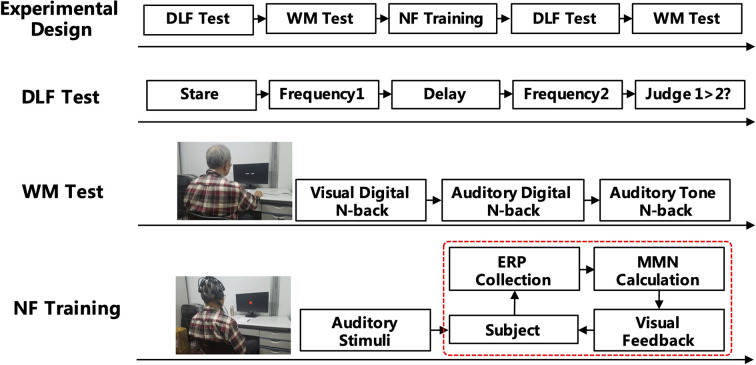
Experimental design of the mismatch negativity (MMN) neurofeedback training.

The experiments included neurofeedback training, memory-based cognitive tests, and auditory frequency discrimination tests before and after the training (see Figure 1). The typical procedure for estimating auditory frequency discrimination was using a frequency increment detection paradigm, where listeners are instructed to compare a reference tone with a series of lower or higher frequency tones (Kishon-Rabin et al., 2004). Memory tests included visual digital N-back, auditory digital N-back, and auditory tone N-back, which are mainly used to test the WM ability of the subjects. The neurofeedback training calculates the amplitude of MMN of the subjects in real time by giving the auditory tone stimulus and performing a visual signal feedback to allow the subjects to independently adjust the amplitude of MMN.

### Auditory Frequency Discrimination Test

In this experiment, 1,000 Hz was used as a standard (reference) tone, with a set of comparison stimuli varying from 500 to 1,500 Hz in 100 Hz steps (e.g., 500 Hz, 600 Hz, 700 Hz, 800 Hz, 900 Hz, 1,100 Hz, 1,200 Hz, 1,300 Hz, 1,400 Hz, and 1,500 Hz). Each subject received auditory stimuli transmitted by a GSI-61 audiometer with binaural presentation *via* headphones (TDH 50) at 65–70 dB HL. The whole experiment was performed using E-Prime (Psychology Software Tools Inc., Pittsburgh, PA, USA). Each subject received 10 sets of pretrials to become familiar with sound stimulation. The actual test lasted for approximately 30 min.

### Working Memory Test

WM refers to the structures and processes used to temporarily store and manipulate information during ongoing processing and distraction. The N-back task is one of the most popular measures of WM in cognitive neuroscience. In a classical N-back task, subjects are presented with a series of stimuli, and the task is to determine if each stimulus is consisted with an Nth stimulus shown previously. By manipulating the value of N, the processing load can be changed systematically, which influences the changes in accuracy and reaction time (RT; Jaeggi et al., 2010). In this experiment, we adopted a visual digital N-back (1-, 2-, and 3-back), an auditory digital N-back (1-, 2-, and 3-back), and an auditory tone N-back (1-, 2-, and 3-back). N-back uses three types of stimuli: visual digits (digits from 1 to 9, except 7), auditory digits (digits from 1 to 9, except 7), and auditory tone (tone frequencies include 200 Hz, 400 Hz, 800 Hz, 1,000 Hz, 1,200 Hz, 2,000 Hz, and 4,000 Hz; Zhang et al., 2016). The digits were recorded from Google with Chinese pronunciation.

### Neurofeedback Training

The neurofeedback training was completed five times in 2 weeks with an interval of less than 3 days and more than 1 day by all participants. Each neurofeedback training contains five training sessions. The standard MMN was induced by a standard stimulus of 1,000 Hz (80%), and the deviant stimulus of 2,000 Hz (20%) was based on the oddball paradigm (Lin Y. et al., 2013). A modification of the MMN neurofeedback training protocol was used in this study (Chang et al., 2014). MMN was elicited by an auditory stimulus based on an oddball paradigm: 1,100 Hz was the standard stimulus; the deviant stimulus was two times the individual auditory discriminant threshold value (Restuccia et al., 2009). The standard stimulus accounted for 80% of the stimuli. The stimuli were presented in a random order. The frontal (Fz), central (Cz), and parietal electrodes (Pz) were used as the training sites.

During each neurofeedback training session, the first MMN amplitude was used as the individual baseline threshold. Then, the amplitude of MMN was updated every 0.5 s. The amplitude of the real-time MMN was fed back as a visual disc signal, and the radius of the disc was proportional to the amplitude of MMN. When the real-time MMN amplitude was greater than the threshold, a red disc appeared; otherwise, a green disc appeared. Subjects were instructed to focus on the visual stimuli and silently count the red discs during training to ignore the auditory stimuli. During the neurofeedback training, the subjects attempted to use their strategies to try to increase the number of appearances of red discs (Pei et al., 2020).

### Data Processing and Statistical Analysis

The results of the auditory discrimination threshold test and the N-back tasks were analyzed by the E-Prime software. The curve fitting of the method is based on a weighted cumulative Gaussian distribution function *f*(*p*), as shown in Equation (1):

(1) $$f(p)=0.5\left[1+\left(\frac{P-PStd.}{\sigma \sqrt{2}}\right)\right]$$

where σ is a parameter describing the steepness of the curve and can be considered as a qualitative measure of the 84% discrimination threshold and standard (1,100 Hz). R^2^ evaluates whether each psychometric function fits a cumulative Gaussian distribution (Wichmann and Hill, 2001a,b; Pei et al., 2020).

The EEG data were analyzed offline *via* EEGLAB, an open-source MATLAB toolbox for electrophysiological signal processing (Delorme and Makeig, 2004). The raw EEG signal underwent 0.5 Hz high-pass and 45 Hz low-pass FIR filters. Independent component analysis (ICA) was used to reject artifacts of the EEG signals, and the main components are responsible for the eye movements and blinks.

Statistical analysis was performed by using SPSS 19 (SPSS, Chicago, IL, USA). Data are expressed as the mean ± standard error. A pointwise paired *t*-test of the standards and deviants was performed from 100 ms to 300 ms of ERP.

The amplitude and latency of MMN during the training were statistically analyzed using a repeated measures analysis of variance (ANOVA) with the “training day” (day 0 to day 5) and “electrode” (Fz, Pz, and Cz). A paired *t*-test was performed to compare the pre/post neurofeedback training (day 0 and day 5). Auditory discrimination accuracy was statistically analyzed using a repeated-measure ANOVA within the assessment time (pre/post training) and “frequency” (500–1,500 Hz in 100 Hz steps). The auditory discrimination threshold and the behavioral results of the WM were analyzed with the paired *t*-test. The significance level was set at *p* < 0.05, and high significance was set at *p* < 0.01. The previously described methods of statistical analysis have been verified by other experimental studies (Zhang et al., 2018).

## Results

We evaluated the standard MMN changes from ERP, auditory frequency threshold, and WM ability by cognitive behavioral tests. The amplitude of MMN was significantly increased with no significant change in latency after 5 days of MMN neurofeedback training at Pz. The accuracy of the subjects in the auditory tone 3-back test was increased significantly.

### ERP Analysis of MMN

**Figure 2:**
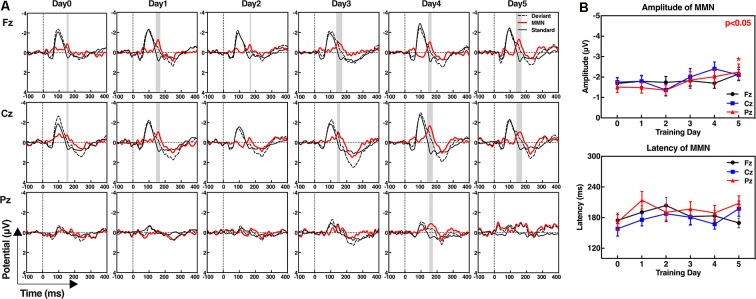
Event-related potential (ERP) waveforms of the standard stimuli, deviant stimuli, and mismatch negativity (MMN; **A**), and the amplitudes and latencies of MMN at frontal (Fz), central (Cz), and parietal electrodes (Pz; **B**). **(A)** MMN waveforms were obtained by subtracting the ERPs in response to the standard stimuli from ERPs in response to the deviant stimuli. The gray shaded areas show the significant differences between the standard and deviant stimuli from 100 ms to 300 ms, *p* < 0.05. **(B)** Paired *t*-test; day 5 compared with day 0, *p < 0.05.

We collected the standard MMN of the subjects before the first training and after each training day. The six ERP waveforms at the Fz, Cz, and Pz electrodes are shown in Figure 2A. The results demonstrate high signal-to-noise ratio of ERP waveforms of the subjects and a more pronounced MMN that appears at the last training (day 5) at all three midline electrodes. Assessment of the amplitude or latency of MMN during the neurofeedback training (from day 0 to day 5) by repeated measure indicates that the “training day” has no major effect (amplitude: *F*_(5,80)_ = 1.230, *p* = 0.303; latency: *F*_(5,80)_ = 1.230, *p* = 0.303). Therefore, the MMN characteristics of the subjects did not change significantly with increase in the number of training days. However, the amplitude of the MMN at the Pz electrodes was significantly improved after 5 days of neurofeedback training according to the paired *t*-test (*t* = 2.232, *p* = 0.040). There was no significant increase of day 5 in the Fz (*t* = 1.054, *p* = 0.308) and Cz values (*t* = 1.307, *p* = 0.210) compared with that observed on day 0. The latency of MMN showed no significant changes at each electrode after five training days (Fz: *t* = 0.358, *p* = 0.725; Cz: *t* = −1.917, *p* = 0.073; and Pz: *t* = −1.815, *p* = 0.088; Figure 2B).

**Figure 3:**
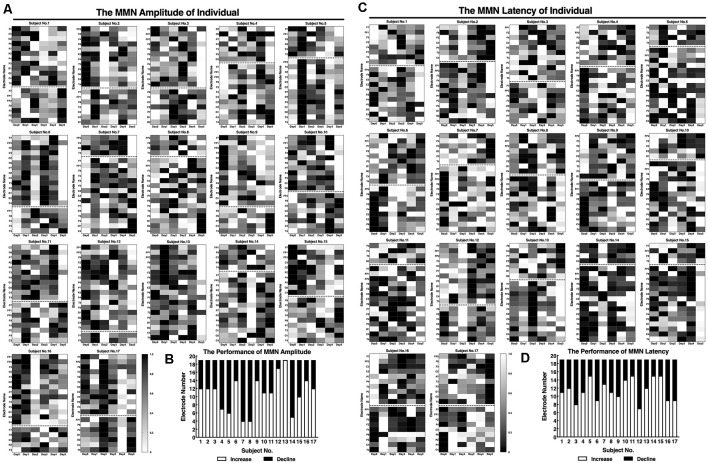
Standard values of individual MMNs before and after the neurofeedback training. The amplitudes **(A)** and latencies **(C)** of the individual MMNs were normalized by processing the true values from 0 to 1. **(A,C)** The maximum value of the amplitude is shown in white, and the minimum is shown in black for each subject. **(B,D)** Statistics of the number of electrodes on day 5 performance amplitudes compared with that on day 0.

Figure 3 shows the amplitudes and latencies of MMN at 19 channels of each subject. In addition to three midline training electrodes, the amplitudes of the MMN at other locations after neurofeedback training were increased, especially in subject No. 13, in whom the whole brain was effectively improved. With regard to latencies, the number of electrodes with reduced signal is almost the same as that of increased signals.

### Auditory Discriminability

The auditory discrimination threshold of an individual was tested before and after the training, and the value before the training was used as the personalization parameter (deviant stimuli) of the MMN neurofeedback protocol.

**Figure 4:**
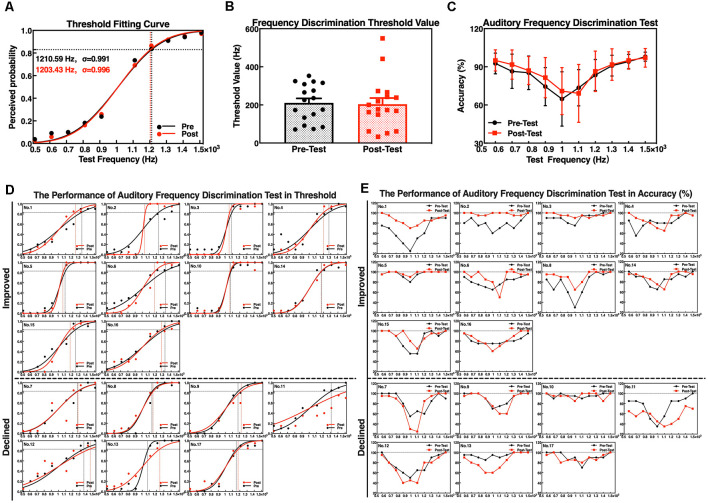
Performance of auditory frequency discrimination task before and after the neurofeedback training. **(A)** Frequency threshold fitting curve; **(B)** discrimination threshold value; **(C)** discrimination accuracy. Individual performance of auditory frequency discrimination test as a threshold fitting curve **(D)** and as accuracy **(E)**.

The results of the threshold fitting curve indicate that the range and gradient of the test frequency in the auditory frequency discrimination test were suitable for the SCD patients; the σ value was almost 1 before (*σ* = 0.991) and after (*σ* = 0.996) the neurofeedback training (Figure 4A). The threshold value was reduced overall after the neurofeedback training (Figure 4B), but the reduction was not significant (*t* = 0.261, *p* = 0.798) due to individual differences (Figure 4D). The average accuracies of 1,100 Hz and 1,500 Hz detection declined; however, detection of all other test frequencies was improved compared to that before the training (Figure 4C). Assessment of discrimination accuracy of test frequency by repeated measure indicates that there is no major effect of assessment time (pre/post neurofeedback; *F*_(1,16)_ = 0.776, *p* = 0.391). There were large individual differences in the accuracy rate, although 10 of 17 participants have improved after the training (Figure 4E).

### Working Memory Performance

Considering the differences in the educational levels and cognition between the participants, we have conducted three types of WM for cognitive assessment, and the auditory tone 3-back test performance was significantly improved in accuracy after the neurofeedback training.

**Figure 5:**
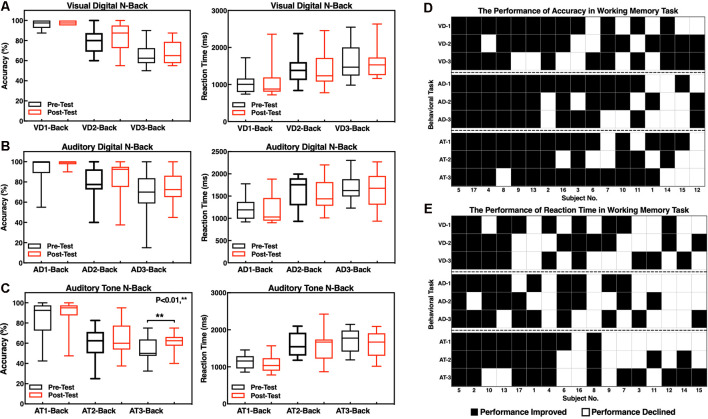
Accuracy and reaction times (RTs) of the N-back tasks. **(A)** Visual digital N-back, **(B)** auditory digital N-back, and **(C)** auditory tone N-back. **p < 0.01; red or black bars above and below the graph show the maximum and minimum accuracy or RT in each task, respectively. The statistics evaluation of the tasks based on the performance of individuals as accuracy **(D)** and RT **(E)**.

The accuracy and RTs of three types of the N-back tasks before and after the neurofeedback training were analyzed by a paired *t*-test. The results showed that the effect of the neurofeedback training was not significant for accuracies of visual digital N-back (1-back: *t* = −1.765, *p* = 0.097; 2-back: *t* = −1.676, *p* = 0.113; 3-back: *t* = −0.841, *p* = 0.413) and auditory digital N-back (1-back: *t* = −1.957, *p* = 0.068; 2-back: *t* = −1.001, *p* = 0.332; 3-back: *t* = −1.437, *p* = 0.170), although the values show an upward trend. However, for the auditory tone N-back, there was a significant improvement in accuracy in auditory tone 3-back (*t* = −2.947, *p* = 0.009); 1-back (*t* = −1.838, *p* = 0.085) and 2-back (*t* = −1.714, *p* = 0.106) showed no significant difference. No significant differences in RTs were found in visual digital N-back (1-back: *t* = −0.616, *p* = 0.546; 2-back: *t* = 0.676, *p* = 0.509; 3-back: *t* = 0.248, *p* = 0.807), auditory digital N-back (1-back: *t* = 0.198, *p* = 0.846; 2-back: *t* = 1.314, *p* = 0.207; 3-back: *t* = 0.258, *p* = 0.799) or auditory tone N-back (1-back: *t* = 1.259, *p* = 0.226; 2-back: *t* = 0.738, *p* = 0.471; 3-back: *t* = 1.318, *p* = 0.206; Figures 5A–C). Individual performance results showed that after the neurofeedback training, the accuracy was improved and the response speed was faster than before in various WM tests; however, most of the results were not statistically significant except the 3-back auditory tone task (Figures 5D,E).

**Figure 6:**
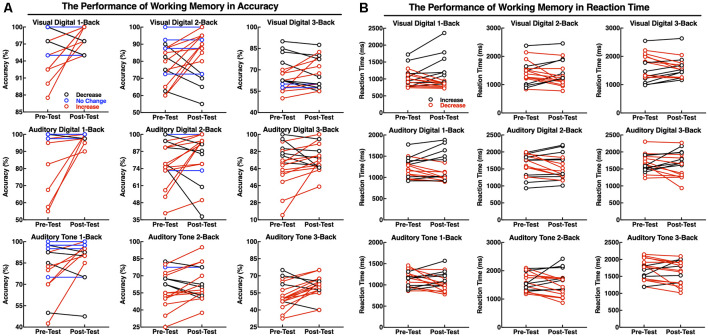
Performance of the individuals in working memory (WM) tasks as accuracy **(A)** and RT **(B)**. The red line indicates that the accuracy was increased or that the reaction speed improved; the black line indicates that the accuracy was deceased and the reaction speed declined; and the blue line indicates no changes in performance accuracy.

The difficulty level of 1-back is the lowest, and thus, most of the participants did not obtain a significant improvement after the training because they had already achieved high scores before the training. The difficulty level of 2-back is medium, and the majority of the subjects clearly improved the accuracy of the tests. The most difficult task is 3-back; 13 subjects improved the accuracy, and the clinical effective rate (the number of improved/the number of non-improved) reached 76.47% (Figure 6A). The RTs of a task were increased concomitant to the difficulty of a task. In other words, the 1-back task is the fastest, and the 3-back task is the slowest. The neurofeedback training had no effect on RTs in 1-back, 2-back, or 3-back tasks (Figure 6B).

## Discussion

In this study, we successfully used an MMN neurofeedback protocol in SCD patients. After five training days during 2 weeks, the training significantly strengthens the amplitude of MMN and increases performance of the patients in WM tests, especially in the auditory tone 3-back tasks. This study is the first to explore the effect of the MMN neurofeedback training on auditory frequency discriminability and enhancement of the working auditory memory in SCD patients by regulation of the MMN activity.

### Unconscious Learning and Personal Intervention Method

Recently, fMRI neurofeedback studies have shown that brain networks can be modified without intention and learning consciousness (Ramot et al., 2016). The activity of the visual cortex can be regulated to influence the visual perceptual learning with no stimulus presenting or the subjective consciousness of the subjects during the neurofeedback training (Shibata et al., 2011; Chang et al., 2014). MMN responses are elicited by any discriminable auditory change that cannot be consciously discriminated. The healthy participants can unconsciously experience a significant improvement in the auditory discrimination of the stimuli applied during a previous study. Each subject was asked to focus on the visual signal (red discs) during our MMN neurofeedback training, and the subjects were required to neglect the auditory stimuli of the experiment as much as possible. Moreover, our results showed that the improvement of the amplitudes of MMN after the training is in agreement with previous studies (Chang et al., 2014). Numerous studies demonstrated that the parietal cortex is consistently linked to and involved in human memory processing, although early studies mostly focused on the medial temporal and frontal lobes (Gilmore et al., 2015). The intrinsic connectivity of the parietal memory network (PMN) reflects the familiarity of the stimuli in memory encoding and retrial and is disrupted in AD (Hu et al., 2019). The Pz electrode may be a potential target site for memory regulation in the neurofeedback training. The latencies of MMN in SCD patients showed no significant differences after training. The individual auditory discrimination threshold was used as the deviation stimulus in our study while a previous study used a fixed frequency as the deviation parameters. Some of our results are inconsistent with the previous data, and the discrepancy may be caused by different paradigm parameters of the experimental design (Chang et al., 2014, 2017). Considering that the MMN index discrimination of various sound stimuli can result from a rigid matching similar to behavioral discrimination, the MMN signals are associated with the magnitude of deviation and involve perceptual discriminability (Näätänen et al., 2017). In SCD patients, auditory discrimination is different because we observed that the auditory frequency discrimination threshold ranges from 1,200 to 300 Hz. A personal protocol to regulate the amplitude of MMN may be more suitable in the clinical neurofeedback training. Therefore, our results generate additional evidence that MMN neurofeedback can be used as an EEG-specific unconscious and personal neurofeedback training in the SCD patients, thus raising a possibility for a clinical intervention even in the case of the states of altered consciousness.

### Improving the Auditory Discriminability

Previous studies in 2014 have shown that MMN neurofeedback training can improve discrimination between very similar tones, such as “1,000 Hz” and “1,008 Hz” by Chang et al. (2014). A study published in 2017 used MMN neurofeedback to successfully discriminate the English pronunciation between the letters “l” and “r” to facilitate the foreign language learning (Chang et al., 2017). These studies focused on the use of learning to discriminate the targeting of the similar stimuli, similar to discrimination between standard and deviant stimuli during the neurofeedback training to elicit MMN. However, in our study, the individual auditory discrimination threshold has to be considered because individual deviant stimuli were used in the training protocol. The thresholds decreased after the training indicating that auditory discrimination is improved; however, this effect was not statistically significant and only 60% of SCD patients (*n* = 10) were successful. We found that the accuracy of those SCD patients, which failed to decrease the auditory discrimination threshold, was lower than the accuracy in the successful tasks; the measurement of the threshold is based on the successful task statistics. Importantly, the subjects who demonstrated good training results have more compact training time. Previous studies indicate that different training intensity will influence the training effect and excessive or insufficient training may actually reduce the effectiveness of the training. Monitoring the learning curves of the subjects to set the appropriate training cycles may be implemented in the future. Moreover, the most successful subjects used the psychological strategies for recalling positive things or focusing on certain things during the neurofeedback training and were able to adjust their psychological strategies autonomously when their training scores were decreased. To improve the efficiency of the intervention, the optimal training intensity and psychological strategy are worth to be explored in the future studies (Enriquez-Geppert et al., 2017).

### Enhancing the Performance of Auditory Working Memory

At present, memory regulation based on the neurofeedback focuses on the regulation of the EEG frequency band. Training the alpha rhythm activity by neurofeedback to increase memory and attention has to consider that alpha activity inhibits the processes that are unnecessary or conflicting with the task being performed and thus can promote attention and memory by actively suppressing the distracting stimuli (Klimesch, 1999). Theta oscillations have been correlated with the memory encoding (Sauseng et al., 2010), and their excess is detected in AD patients; regulation of the theta activity always involved a neurofeedback protocol in the healthy subjects to enhance memory, even in the MCI, AD, and dementia patients (Luijmes et al., 2016). Previous studies have shown that in schizophrenic patients, MMN impairment is accompanied by poor WM performance (Kiang et al., 2007); a similar association is observed in the case of WM impairment in alcoholics and low performance of MMN (Bonetti et al., 2018). WM is considered a cognitive system, which is responsible for operating the information of various sensory modalities; and the neurons of the pre-supplementary motor area encode tactile and auditory information involved in the WM tasks, thus using a shared representation for both sensory modalities. A recent study indicated that individuals with higher visual WM performance have increased automatic neural responses to changes in the auditory characteristics (Bonetti et al., 2018). In older adults, automatic auditory processing guided by selection history was largely lacking (Sur and Golob, 2019). From the perspective of neurobiology, the generation of MMN is associated with N-methyl-D-aspartate receptors (NMDRs), which is the major molecular device for regulating the plasticity of the synaptic and memory functions (Li and Tsien, 2009). NMDRs are crucial in the activity-dependent synaptic changes, learning, and WM due to mediation of the excitatory postsynaptic potentials (Baez et al., 2018; Lisman et al., 1998). Moreover, auditory mismatches engage a hierarchical functional network of cortical sources, which are also interconnected by auditory white matter pathways. Neuroimaging studies have found that a disrupted pattern of the AD connectome that starts in peripheral regions, which refer to a set of cortical regions weekly connected in brain structural networks, and then hierarchically propagates to highly connected cortical brain regions, when patients show clinical symptoms. Moreover, peripheral regions might contribute to impaired memory performance in patients with SCD (Wang et al., 2017; Yan et al., 2018). Neurofeedback training can improve the auditory cortex plasticity by regulating neuronal synaptic plasticity and changing the neural networks of the brain (Emmert et al., 2017). Neurofeedback is a safe and noninvasive intervention that can influence cognitive functions and behavioral changes. To date, no adverse reactions of the neurofeedback training have been reported; however, it is not recommended to train people with serious mental problems (Marzbani et al., 2016). Therefore, enhancing WM based on the MMN neurofeedback may be an effective method of intervention not only in SCD patients but also in healthy older people; however, the persistence of the training effects lacks the data of the clinical follow-up study.

In this study, we have designed three types of WM in the form of N-back with three types of levels to assess the effects of the training to account for the degree of completion of the tasks in SCD patients with various educational backgrounds. The results indicate that the accuracy scores were improved in all WM tests vs. baseline and especially in the auditory tone 3-back, which significantly increased after the training. The auditory tones are the stimuli to induce MMN; thus, there is a limit of the performance of the WM effect with regard to the visual and auditory digits. Previous studies have shown an impact of the stimulation task type training on the WM types (Chan et al., 2019; Linares et al., 2019). A behavior study showed that enhanced transfer of the auditory discrimination learning is mediated by WM, which reflects that WM updating is related to fine auditory discrimination (Zhang et al., 2016). Actually, SCD is not strongly correlated with the concurrent level of cognitive ability as measured by objective cognitive tests; previous studies have shown that subjective memory complaints are associated with subsyndromal or subclinical depression in community-dwelling older adults (Zlatar et al., 2018). Therefore, the targeting of specific interventions for the regulation of cognitive ability will be more helpful in the case of the intervention treatment of patients with heterogeneous SCD.

### Limitations and Further Research Directions

To assess the effectiveness of the clinical intervention, certain issues deserve further attention. For experimental design, setting up a double-blind experiment with a strict control group will be more convincing to demonstrate the effect of the training. Given the heterogeneity of patients, in addition to SCD heterogeneity, to include differences in the learning ability and living conditions, recruitment of a control group with similar subject characteristics is difficult considering that post-training activities that influence training results cannot be controlled. Current clinical intervention studies focus on small samples and the desired sample should be as large as possible. The timeline of the assessment of the clinical scales is usually longer than 3 months; hence, the methods and tasks for accessing the short-term intervention need to consider the operability and effectiveness of the patients. Moreover, we focused on discussion of the individual results while explaining the average results. Given the importance of SCD in diagnosis of the neurocognitive disorders and as potential predictor of a future cognitive decline, the individualization and targeted treatment of intervention methods will be explored in the future studies.

We propose a novel neurofeedback protocol based on the MMN amplitude to improve the performance of auditory WM in SCD patients. The preliminary results of our study may indicate that neurofeedback training based on MMN may be a potential treatment for the elderly with aging memory decline.

## Data Availability Statement

The datasets for this article are not publicly available because the research in this article involves personal information from SCD patients. Request to access the datasets should be approved by the Medical Research Ethics Committee and Institutional Review Board of Xuanwu Hospital.

## Ethics Statement

The studies involving human participants were reviewed and approved by Medical Research Ethics Committee and Institutional Review Board of Xuanwu Hospital. The patients/participants provided their written informed consent to participate in this study. Written informed consent was obtained from the individual(s) for the publication of any potentially identifiable images or data included in this article.

## Author Contributions

GP contributed for the interpretation of data and wrote the article. RY and ZS designed the experiments and verified the effectiveness of the experiment. GG, YQ, and SW contributed to the collection and analysis of the data and performed the experiments. ML and JW are mainly responsible for the conception and design of the work. RG, YH, and TY revised the work critically for important intellectual content and also gave final approval of the version to be published.

## Conflict of Interest

The authors declare that the research was conducted in the absence of any commercial or financial relationships that could be construed as a potential conflict of interest.
